# Evaluation of the Expression of IDO and PTEN in Human Kidney Cancer

**DOI:** 10.3390/cimb47050359

**Published:** 2025-05-13

**Authors:** Gábor Kónya, Zsuzsanna Szabó, Nikoletta Dobos, József Király, Krisztián Szegedi, Anna Vass, Ákos Steli, Csaba Szász, Balázs Dezső, Barbara Zsebik, Gábor Halmos

**Affiliations:** 1 Department of Biopharmacy, Faculty of Pharmacy, University of Debrecen, 4032 Debrecen, Hungary; konya.gabor@pharm.unideb.hu (G.K.); szabo.zsuzsanna@pharm.unideb.hu (Z.S.); dobos.nikoletta@pharm.unideb.hu (N.D.); kiraly.jozsef@pharm.unideb.hu (J.K.); vass.anna@pharm.unideb.hu (A.V.); steli.akos@pharm.unideb.hu (Á.S.); zsebik.barbara@pharm.unideb.hu (B.Z.); 2 Doctoral School of Pharmaceutical Sciences, University of Debrecen, 4032 Debrecen, Hungary; 3 Department of Urology, Faculty of Medicine, University of Debrecen, 4032 Debrecen, Hungary; szegedi.krisztian@med.unideb.hu; 4 Department of Pathology, Faculty of Medicine, University of Debrecen, 4032 Debrecen, Hungary; szasz.csaba@med.unideb.hu (C.S.); bdezso@med.unideb.hu (B.D.)

**Keywords:** IDO, PTEN, ccRCC, A-498, CAKI-2

## Abstract

Immunotherapy has become one of the primary forms of cancer treatment. The inhibition of immune checkpoint molecules, including indoleamine 2,3-dioxygenase (IDO), is a promising approach for immunotherapy. Phosphatase and tensin homolog (PTEN) is well known as a tumor suppressor that antagonizes oncogenic signaling molecules/pathways and plays a key role in the prognosis and (immuno)therapy of the disease. In this study, twenty healthy and tumorous renal tissue pairs were investigated, and the mRNA (RT-qPCR) and protein (Western blot) expression of IDO and PTEN were analyzed. In two cancer cell lines (CAKI-2; A-498), the protein of IDO and PTEN was measured followed by IDO induction with interferon alpha-2 (IFN-α2). According to our results, a significantly higher mRNA expression of IDO and PTEN was found in tumorous tissues compared to the adjacent healthy kidney specimens. The mRNA expression of IDO and PTEN showed a positive correlation in 80% of the sample pairs. Western blot results confirmed the protein expression of both IDO and PTEN. In the cell lines, immunocytochemistry showed that IDO is inducible with IFN-α2. In summary, our results suggest that IDO expression may play a role in the development of renal cancer, and IDO as well as PTEN might be potential biomarkers for patients with RCC.

## 1. Introduction

Renal cell carcinoma (RCC) is a urological type of cancer, and its occurrence is 5% in men and 3% in women in relation to all cancers [1,2]. In most cases, it has no symptoms, and renal masses are mostly revealed accidentally during ultrasound or CT examinations, which are carried out due to other clinical symptoms [1,2]. Although RCC is not the most frequent urological cancer type, it has a mortality rate of around 25% [3]. Metastases are present in 20–30% of the patients at the time of diagnosis, which accounts for the high mortality rate [4]. The most common type of RCC is clear-cell renal cell carcinoma (ccRCC), representing more than 75% of all RCC cases. The other two significant types of renal carcinoma are papillary and chromophobe RCC [5]. Only a few risk factors are known, such as smoking, exposure to cadmium, or Von Hippel–Lindau disease [5]. The major genetic changes that lead to the development of ccRCC are the inactivation of the Von Hippel–Lindau gene and other genetic mutations that frequently affect the phosphatase and tensin homolog (PTEN) [6]. The first-line treatment options for RCC are radical nephrectomy, partial nephrectomy, or cytoreductive nephrectomy, with a 5-year survival rate of 85%. However, in those cases where metastasis has already been observed or the surgical removal of the organ is not possible, the only option is drug therapy [7]. This cancer type has an inadequate response to chemotherapy mostly because the proximal-tubule cells express multidrug-resistant exporters [8]. In the last few years, the chances of survival have increased in these cases as a result of the development and availability of new immunotherapies [9,10,11,12,13,14,15].

Tumor biomarkers help in the development of fast, cost effective, and efficient therapies. These biomolecules can be found in the tumor microenvironment, blood, or in any bodily fluid [16]. From the past more than one hundred years, plenty of tumor markers were discovered, and now these are in human diagnostics and medicine. Alpha-fetoprotein (AFP) is used in clinical screening and can help in the evaluation of hepatocellular carcinoma (HCC) [17]. Another commonly known biomarker is the P53 oncogene, and its mutation often occurs in various cancer types [18]. Tumor necrosis factor-α (TNF-α) is an inflammation-related regulatory protein that is increased in multiple tumor types such as melanoma, prostate cancer, or RCC. Carbonic anhydrase 9 (CA9) is a protein biomarker which can predict the aggressivity of RCC. Elevated serum concentrations of CA9 in patients with metastatic ccRCC can reduce the overall survival [19]. Mucin-1 (MUC1) transmembrane protein overexpression supports the development of various tumors such as liver cancer, colon cancer, or RCC. The high level of MUC1 increases the resistance to various treatments in RCC cases, and this biomarker could provide support in choosing the best therapy for patients [20]. Various microRNAs (miRNAs) could also serve as biomarkers. The overexpression of miR-21 and miR-221 is a prognostic factor in RCC and indicates poor survival of patients [21].

Indoleamine 2,3-dioxygenase (IDO) is a cytosolic heme-containing enzyme and is the key regulatory enzyme in tryptophan metabolism as it is responsible for the conversion of tryptophan into kynurenine [22]. IDO also has an immunomodulating function by affecting this metabolic pathway; it suppresses immune activation cells and promotes immunosuppressive cells, playing a role in poor survival in various cancer patients. IDO is able to modulate naive T cells and promote their differentiation into regulatory T cells [23]. The IDO gene family encodes not only IDO but also indoleamine 2,3-dioxygenase-2 (IDO2) and tryptophan-2,3-dioxygenase (TDO). IDO2 is highly tissue-specific and mostly located in the placenta and liver, while TDO is expressed in the liver and in the brain [24,25]. IDO expression can be triggered by interferon gamma (IFN-γ), which is usually produced by T cells. Like other cytokines such as IL-10 and IL-27, TGF-β can also increase the level of IDO [26]. Although IFN-γ is one of the most potent inducers of IDO, IFN-α has a similar effect in various cell types [27]. In different types of cancer, mRNA and protein for IDO were overexpressed compared to healthy tissues. Elevated IDO expression is well known in cervical cancer or in brain tumors; moreover, IDO overexpression in the brain leads to depressive-like behavior [28].

It has been discovered that the loss of IDO or its decreased activity leads to autoimmune diseases due to its immunoregulatory function [29]. IDO could also work as a prognostic biomarker for multiple cancer types. Brandacher showed that high IDO levels support colorectal cancer progression and have a negative effect on survival rate [29]. Increased IDO has a negative effect on the CD3+ T cells and correlates with the chance of liver metastases [30]. In liver hepatocellular carcinoma, increased IDO expression was detected in tumor samples compared to healthy tissues. Moreover, IDO shows grade and stage dependency too [31]. IFN-γ-induced IDO activity has multiple effects, which can be analyzed in cellular models. Hepatocellular models showed that IDO increased the aryl hydrocarbon receptor (AhR) production, which stimulates proto-oncogene tyrosine-protein kinase Src (Src) activity and works as a PTEN inhibitor [31]. Previous studies have reported that an increased mRNA level of IDO correlates with an increased protein level of IDO in tumorous tissues compared to healthy tissue samples [32]. According to previous results, IDO could serve as a prognostic factor in primary tumors, and its high expression level could indicate a worse prognosis of the disease [32]. Nivolumab is a commonly used therapeutic agent in metastatic RCC cases. Recent results suggest that IDO is mostly expressed by the tumor endothelial cells, and IDO overexpression was observed within the responders group [33]. Indoleamine 2,3-dioxygenase 1 (IDO1) was found at the plasma membrane level of various tumor cells. However, further studies are necessary to determine the function and its subcellular localization of IDO in pathological conditions, such as cancer [34].

The phosphatase and tensin homolog (PTEN) gene is located on chromosome 10q23.3 [35]. PTEN localizes in the plasma membrane and in the cytosol and nucleus as well as subcellularly in the mitochondria, endoplasmic reticulum (ER), and mitochondria-associated membranes (MAMs) [36]. This gene and its encoded intracellular protein have been highly investigated in the last few decades as it has a key role in cell division, growth, and apoptosis [37]. Moreover, PTEN can inhibit the PI3K/PTEN/AKT signaling pathway, which is essential in healthy cell division; thus, PTEN has a role in preventing cancer development. This means that the loss or mutation of PTEN could be a first step towards carcinogenesis [38,39]. PTEN loss or mutation can be related to PTEN hamartoma tumor syndrome, such as in Cowden syndrome. Individuals with these syndromes have a high risk to develop multiple benign hamartomas or breast or endometrial cancers, and based on recent case study reports, the loss of PTEN function also increases the risk of RCC [25]. Although PTEN is one of the novel topics in oncology, it is still unclear whether it could be a prognostic factor in ccRCC cases.

Only a few percent of RCC cases develop PTEN mutations, which usually results in the loss of PTEN function [40]. Chaoyang Zhu et al. found a correlation between PTEN expression and survival rate [41]. PTEN expression suggests a better survival rate and life expectancy than the loss or defective mutation of PTEN [41]. PTEN expression also has a therapeutic importance in kidney cancer. PTEN knock-out cell lines showed a highly increased resistance to sunitinib/sorafenib treatment and the promotion of spheroid formations. In metastatic RCC cases where sunitinib and sorafenib have been therapeutically used, 23% of the patients showed dysfunctional PTEN [42].

New biomarkers can not only help in establishing an accurate diagnosis but also offer more targeted therapeutic options. Understanding the relationship between IDO and PTEN can certainly be important in setting up an accurate diagnosis and therapeutic options for kidney tumors. In our study, we aimed to analyze the expression of IDO and PTEN in human RCC tissue samples and cell lines. The implications of the intracellular localization of IDO and PTEN regarding their potential application as cancer biomarkers require additional studies in the future.

We analyzed the mRNA and protein expression of IDO and PTEN in ccRCC tissue samples and in human experimental kidney cancer cell lines. Furthermore, IDO was also induced by IFN-α2 in human kidney cancer cell lines. To the best of our knowledge, this is the first study where PTEN and IDO co-expression is investigated in ccRCC cases.

## 2. Materials and Methods

### 2.1. Human Kidney Tissue Samples and Cell Lines

In the present study, twenty (20) human kidney cancer tissues and adjacent healthy paired tissues were examined. Samples were obtained from patients diagnosed with renal cancer and collected at the Department of Urology, University of Debrecen, Hungary. All available clinicopathological data of the patients, such as age, tumor type, diagnosis and gender, are collected in Table 1. The local Institutional Ethics Committee approved the collection and use of these specimens for the current study (UDE REC/IEC 4831-2017), and informed consent was obtained. The human tissue samples were registered after the surgical removals and stored at −80 °C until further examinations. Histological examination of the samples was observed by an expert pathologist. Tumors were staged using the TNM staging system of the Union for International Cancer Control. Histological grade was determined according to World Health Organization criteria [43].

From the collected samples, 15 human kidney cancer samples were classified as ccRCC. Two oncocytoma cases and two cases of the papillary type of RCC were identified, and one sample originated from a case of angioleiomyolipoma. Distribution of the samples by gender was not equal. A total of 7 tissue samples originated from males, and 13 tumorous samples were isolated from female patients. The age range of the studied population was between 49 and 95 years, while the mean age was 67 years, and the median age was 66 years.

CAKI-2 and A-498 human renal cell carcinoma cell lines (clear-cell renal cell carcinoma histological type) originated from the American Type Culture Collection (ATCC) (Rockville, MD, USA). The cells were grown at 37 °C under a controlled environment, where the CO_2_ and humidity were 5% and 95%, respectively. Iscove’s Modified Dulbecco’s Medium (IMDM) was used as a culturing medium for the cultivation of both human kidney cancer cell lines. It was supplemented with antibiotics (100 U/mL penicillin and 100 µg/mL streptomycin) and 10% Fetal Bovine Serum (FBS).

### 2.2. RNA Isolation

Tissue sample weights were measured, and 20–30 mg of each of the tissue samples used in this study was homogenized using a Blade-type Homogenizer Tissue Ruptor (Ultra-Turrax tissue homogenizer; IKA Labortechnik, Staufen, Germany). For isolation of total RNA from cell lines, A-498 and CAKI-2 cells were collected with centrifugation. The NucleoSpin RNA/Protein commercial kit (Macherey-Nagel, Düren, Germany) was used according to the manufacturer’s instruction in order to isolate RNA and protein from human kidney cancer tissues and human kidney cancer cells. The RNA concentration and purity were measured by a NanoDrop ND-1000 spectrophotometer (Nanodrop Technologies, Wilmington, DE, USA). An OD260/280 ratio around 2.0 was assumed to be an indicator of good RNA quality. RNA samples were also measured at 260/230 nm, and an optical density of 2.0 was considered suitable for gene expression analyses. Until further molecular biological analyzes, total RNA was stored at −80 °C.

### 2.3. Reverse Transcription PCR

The isolated total RNA was transcribed into complementary DNA (cDNA) using an RT-PCR method. The Tetro cDNA Synthesis Kit (BIO-65043, BIOLINE, London, UK) was used for transcribing total RNA into cDNA using a reverse transcriptase enzyme, as instructed by the kit’s manufacturer. In order to start the transcription of 500 ng of cDNA, we first produced a premix for which the quantities for the reagents were listed in the kit’s instructions. According to the protocol, 1 µL of random hexamer, 1 µL of 10 mM dNTP mix, 4 µL of µL 5× RT buffer, 1 µL of RiboSafe RNase Inhibitor, and 1 µL of Tetro Reverse Transcriptase were used in 20 µL of final volume; (20 − (n + 8) µL) DEPC-treated water was added, where n represents the determined RNA concentrations, and the amount of total RNA used in the procedure was calculated. In PCR tubes, the premix and RNA samples were mixed on ice to avoid RNA degradation. The PCR tubes were carefully suspended and placed into the PCR machine (C1000 TM Thermal Cycler, Bio-Rad Laboratories Inc., Hercules, CA, USA). The samples were initially incubated for 30 min at 45 °C. Prior to reverse transcription, the RNA and primer hybridize during the process. The mRNA template and cDNA then underwent a 5 min incubation at 85 °C, during which time they separated from one another before the mixture was cooled on ice. cDNA was stored at −20 °C.

### 2.4. Quantitative Real-Time PCR

Real-time PCR was performed with iTaq™ Universal SYBR^®^ Green Supermix using a CFX-96 Real-Time System (Bio-Rad Laboratories Inc., Hercules, CA, USA), where the final volume was 20 µL. The RT-PCR reaction was performed with IDO and PTEN gene-specific primers (IDO forward: 5′-gccagcttcgagaaagagttg-3′; reverse: 5′-atcccagaactagacgtgcaa-3′; PTEN forward: tggattcgacttagacttgacct-3′; reverse: 5′-ggtgggttatggtcttcaaaagg-3′). At the first qRT-PCR step, each well was heated to 95 °C for 10 min. Forty-five cycles were repeated, where the denaturation step was 15 s long, followed by a 1 min cycle at 60 °C. The final calculation was performed with the ΔΔCt method and GAPDH (forward: 5′-tgtagttgaggtcaatgaaggg-3′; reverse: 5′-acatcgctcagacaccatg-3′) was used as a housekeeping gene.

### 2.5. Statistical Analysis

All qRT–PCR experiments were performed at least three times. Statistical significance was calculated with GraphPad Prism 5.0 (GraphPad Software Inc., San Diego, CA, USA) with a two-way ANOVA test with the Sidak multiple comparison test. Differences were considered statistically significant at a *p*-value ≤ 0.05. Data are expressed as the mean of the biological replicates ± the standard error of the mean (SEM).

### 2.6. Western Blotting of Human Tissue Samples and Cell Lines

Human kidney cancer tissue samples were homogenized with tissue homogenizer (Ultra-Turrax tissue homogenizer; IKA Labortechnik, Staufen, Germany) and then lysed in protein lysis buffer (M-PER, Thermo Fisher Scientific, Waltham, MA, USA), supplemented with protease and phosphatase inhibitors.

The human kidney cancer cell lines CAKI-2 and A-498 were used for further Western blot analyses. Cells were lysed in protein lysis buffer (M-PER, Thermo Fisher Scientific, Waltham, MA, USA), supplemented with protease and phosphatase inhibitors. The quantification of the protein level of the cell lines and tissue samples was performed using Bicinchoninic Acid (BCA) reagent (Thermo Fisher Scientific, Waltham, MA, USA). After dilution with 4× Laemmli buffer, samples were boiled for 8 min at 95 °C, then 40 µg of proteins were loaded on 10% sodium dodecyl sulfate–polyacrylamide gel and separated by electrophoresis (SDS-PAGE). The Precision Plus Protein Dual Color Standard (Bio-Rad Laboratories Inc., Hercules, CA, USA) ladder was used as a molecular weight marker. After the electrophoresis, the proteins were transferred from the gel to a polyvinylidene fluoride (PVDF) membrane. Blotting membranes were blocked with 5% milk-TBS-Tween for 1 h at room temperature and then incubated overnight with the specific primary antibodies at 4 °C (IDO, D5J4E in 1:1000 dilution, BOSTER Biological Technology, Pleasanton, CA, USA; PTEN (D4.3) XP(R) Rabbit mAB, in 1: 1000 dilution, Cell Signaling Technology, Danvers, MA, USA). Horseradish peroxidase (HRP)-tagged anti-mouse IgG or anti-rabbit IgG were used as secondary antibodies (1:3000 dilution, Thermo Fisher Scientific, Waltham, MA, USA). Bands were detected by a ECL chemiluminescence detection kit (Bio-Rad Laboratories Inc., Hercules, CA, USA), and the intensities of the bands were normalized to Hypoxanthine Phosphoribosyltransferase (HPRT) (1:1000 dilution, Sigma-Aldrich, St. Lous, MO, USA).

### 2.7. Immunocytochemistry of Interferon-α2 Treated Cell Lines

IFN-α2 (MedChemExpress, Monmouth Junction, NJ, USA) was used for the induction of IDO in CAKI-2 and A-498 human kidney cancer cells. The drug was applied for 72 h at a final concentration of 1000 U/mL in the cell culturing media [44].

Cells were cultured in cell culture-treated coverslips placed into 6 well plates. The cells were fixed with methanol, washed with PBS, and then permeabilized using 0.1% Triton X-100. The coverslips were incubated overnight at 4 °C with IDO (IDO, D5J4E in 1:100 dilution, BOSTER Biological Technology, Pleasanton, CA, USA) antibody in 1% BSA-0.1% Triton X-100 solution. The next day, the slides were washed 3 times with 0.05% Triton X-100 solution and incubated with Alexa 488-conjugated secondary mouse or rabbit antibodies (1:300, Thermo Fisher Scientific, Waltham, MA, USA) diluted in 2% BSA-0.1% Triton X-100 solution. The slides were washed 3 times and fixed using 1% paraformaldehyde solution. Then, 0.5 µg/mL DAPI (SERVA Electrophoresis GmbH, Heidelberg, Germany) was added for nuclear staining. The fluorescent signal was detected using a ZEISS Axioscope fluorescent microscope (ZEISS, Oberkochen, Germany). Image analysis was performed with ImageJ Fiji 1.53t using the Region of Interest (ROI) Manager function.

## 3. Results

### 3.1. Expression of IDO in Human Kidney Tissues

**Figure 1:**
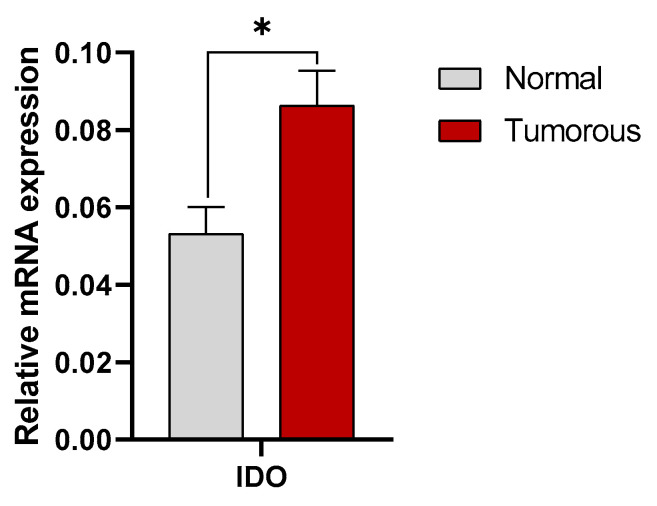
qPCR analysis of mRNA expression for IDO in renal cancer tissues. Mean value of the 20 analyzed normal and RCC tissue sample pairs. For each PCR measurement, 40 ng of cDNA was used, and GAPDH served as the housekeeping gene. Two-way ANOVA with Sidak multiple comparison test was used for statistical analysis. Statistically significant differences were observed between the two groups (*p* = 0.0261). * shows significant difference between the tumorous and normal tissues samples.

IDO expression at the mRNA level was measured in 20 tumorous and healthy tissue sample pairs. According to the quantitative real-time PCR results, RCC tissue samples showed a significantly higher mRNA expression of IDO compared to the adjacent healthy kidney tissues. The statistical analysis performed with two-way ANOVA with the Sidak multiple comparison test showed significant differences (*p* = 0.0261) (Figure 1).

**Figure 2:**
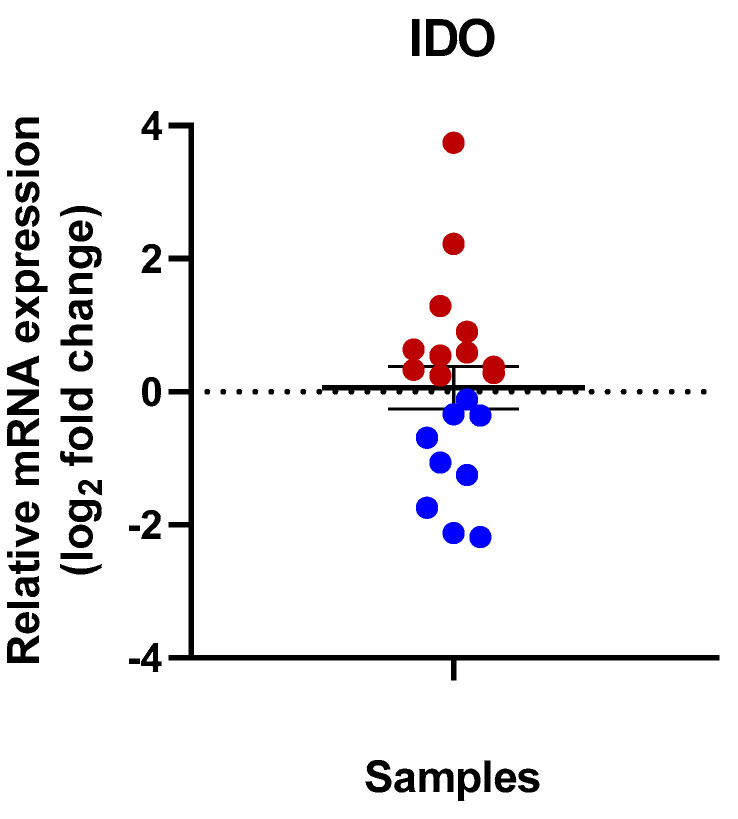
Relative mRNA expression of IDO in human RCC tissue samples. The IDO mRNA level was normalized to the GAPDH housekeeping gene. The mRNA levels of the RCC samples were compared to the adjacent healthy tissue samples; then, the log_2_ fold change was displayed. A total of 11 samples show upregulation (above the 0 line, red dots) of the IDO in the RCC tissue samples. Blue dots show the group of the downregulated samples for IDO of the studied samples.

IDO expression at the mRNA level was measured in 20 tumorous and healthy tissue sample pairs. In 11 samples, a relative upregulation of IDO was detected in the tumorous sediments compared with the normalized IDO mRNA level in the healthy tissue pairs. However, the relative IDO expression was lower in nine cases in the RCC tissue samples compared to the adjacent healthy tissue samples (Figure 2).

In a small group of the studied tissue samples, the expression of IDO at the mRNA level showed relatively high overexpression. As an example, one tissue sample originated from an 83-year-old female patient diagnosed with ccRCC (Grade 2, pT1a of TNM) and showed an extremely high IDO level at the mRNA level; a ~13.4-fold increase was measured in the dissected tumorous part of the tissue compared to the healthy side (Table 2).

In a 76-year-old female patient diagnosed with Grade 2 ccRCC, there was a ~2.45-fold increase in the expression of IDO in the tumorous section compared to the adjacent healthy sample; and another ~4.65-fold IDO overexpression was also detected in a 65-year-old patient with a Grade 3 ccRCC tumor (Table 2).

Another group of the analyzed cancer tissue samples (7 tissues) showed only a slight IDO mRNA increase (smaller than ~2-fold). In this group, there was no correlation between the patients age, sex, and pathological grade of the tumor (Figure 2).

In nine sample pairs, a slight decrease in IDO expression was detected. Three samples showed a ~2-fold decrease at the mRNA level. The highest decrease was detected in a tumorous sample that originated from a 68-year-old male patient (papillary type of RCC, Grade 2 and pT1a). The healthy paired tissue of this sample showed a ~4.6-fold higher mRNA level compared to the tumorous tissue (Table 2) (Figure 2).

### 3.2. Expression of PTEN in Human Kidney Tissues

**Figure 3:**
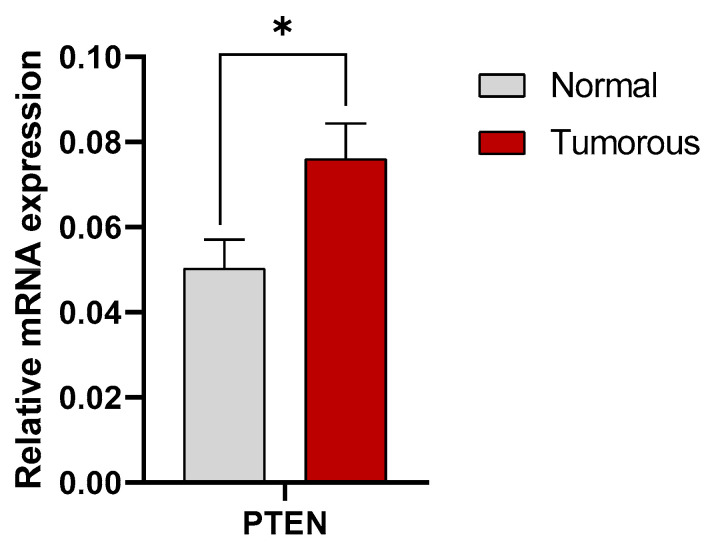
qPCR analysis of mRNA for PTEN expression in renal cancer tissues. Mean value of the 20 analyzed normal and RCC tissue sample pairs. For each PCR measurement, 40 µg of cDNA was used, and GAPDH served as the housekeeping gene. Statistical significancy was observed between the healthy and tumorous samples using the two-way ANOVA test with Sidak multiple comparison test (*p* = 0.0271 for PTEN). * shows significant difference between tumorous and normal tissue samples.

In our study, expression of mRNA for PTEN showed a significantly higher level in tumorous kidney cancer tissues compared to the adjacent healthy tissues. Statistical analyses of two-way ANOVA with Sidak multiple comparison tests show significant differences (*p* = 0.0271 for PTEN) (Figure 3).

**Figure 4:**
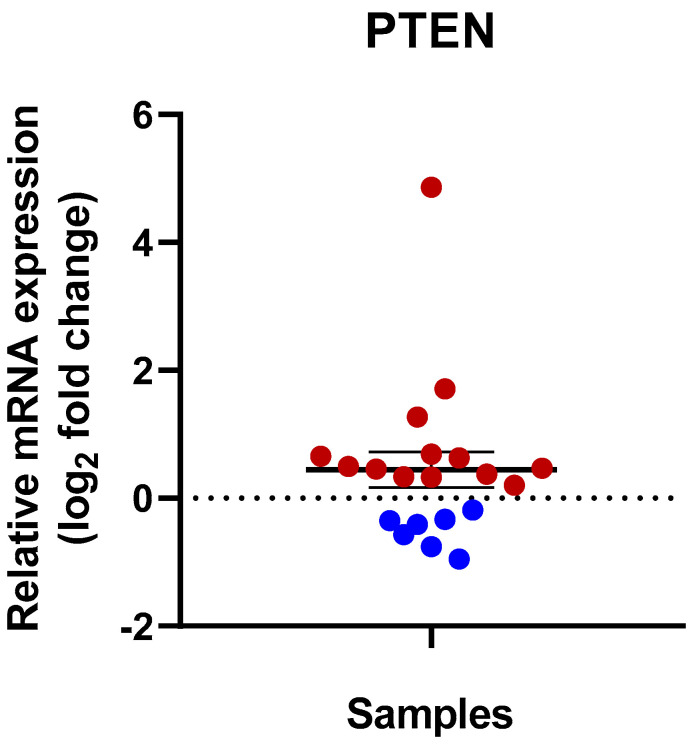
Relative mRNA expression of PTEN in human RCC tissue samples. The IDO mRNA level was normalized to the GAPDH housekeeping gene. The mRNA levels of the RCC sample were compared to the adjacent healthy tissue samples; then, the log_2_ fold change was displayed. A total of 13 samples show upregulation (above the 0 line, red dots) of PTEN in the RCC tissue samples. Blue dots show the group of the downregulated samples for PTEN of the studied samples.

As the quantitative real-time PCR results showed, 13 kidney cancer tissue samples showed a relative PTEN gene expression upregulation (Table 2). In the sample isolated from an 83-year-old woman with Grade 2 ccRCC, PTEN showed an extremely high overexpression with a ~31-fold increase. This was the same sample where the highest IDO mRNA expression was measured (Figure 4).

A tumorous tissue sample obtained from a 65-year-old man (patient number 15) with RCC showed high expression of PTEN (~3.0-fold). In a tumorous sample isolated from a 52-year-old woman with Grade 3 ccRCC and in the pT1b clinicopathological stage, the overexpression of PTEN was considerable: 3.25-times higher than in the adjacent normal tissue. In other patients, the PTEN overexpression in the tumorous pairs was under ~2-fold. Somewhat lower PTEN expression was detected only in seven sample pairs.

Analyzing the co-expression of IDO and PTEN, the following was observed: 10 sample pairs showed increases, while 6 cases showed decreases of IDO and PTEN expression in the tumorous tissue sediments compared to their healthy tissue sample pairs. All in all, 16 sample pairs out of the 20 showed positive correlations between the mRNA expression of IDO and PTEN. In four samples, no positive correlation could be observed between the expression patterns of PTEN and IDO mRNA (Table 2).

### 3.3. Protein Expression of IDO and PTEN

**Figure 5:**
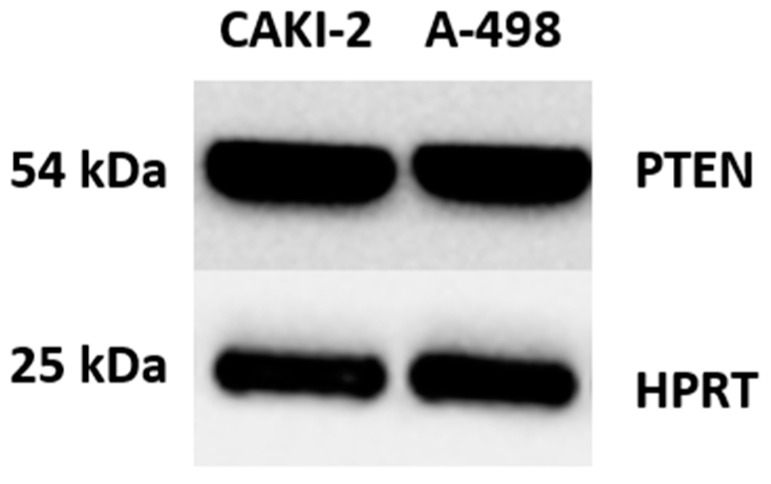
Western blot analysis of the expression of protein for PTEN in CAKI-2 and A-498 cell lines. HPRT was used as a housekeeping protein. A total of 40 µg of proteins were loaded into polyacrylamide gel and separated by electrophoresis (SDS-PAGE); then, protein for PTEN was detected with a specific monoclonal antibody (PTEN (D4.3) XP(R) Rabbit mAB, Cell Signaling Technology, Danvers, MA, USA).

PTEN and IDO protein expression was analyzed in both the A-498 and the CAKI-2 cell lines by Western blotting. There was no significant difference in the PTEN protein expression between the two cell lines (Figure 5). In contrast, none of the cell lines showed measurable levels of IDO protein.

**Figure 6:**
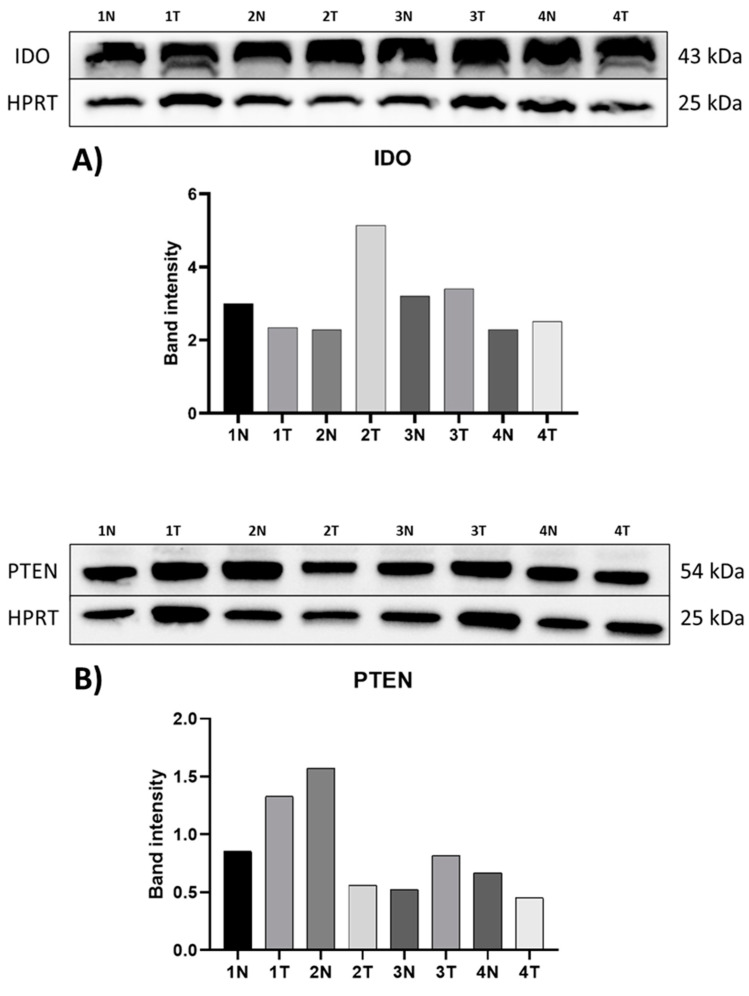
A representative figure of the expression of protein for IDO (**A**). and PTEN (**B**) in adjacent healthy (1N; 2N; 3N; 4N) and tumorous (1T; 2T; 3T; 4T) ccRCC tissue samples. The bands 1, 2, 3, and 4 representing the protein expression of IDO and PTEN are identical to patient numbers 10, 11, 5, and 3, respectively. A total of 40 µg of each protein sample isolated from tissue samples were loaded onto polyacrylamide gel and separated by electrophoresis (SDS-PAGE). A specific monoclonal antibody was used for PTEN (PTEN (D4.3) and IDO (IDO, D5J4E). The intensity of the protein bands was quantified using the Image Lab software (version 5.2.1, Bio-Rad Laboratories Inc., Hercules, CA, USA). (**A**): According to the band intensities, the first sample pair shows downregulated IDO expression, while in the other samples IDO was upregulated in the tumorous tissue sample compared to the healthy ones. (**B**): PTEN was upregulated in the tumorous samples compared to the healthy ones in the first and third samples, while in sample two and four PTEN expression was downregulated.

The protein expression of IDO and PTEN was also studied by Western blotting in representative human kidney cancer samples and their adjacent healthy tissue samples. PTEN protein detected with monoclonal antibody with HPRT housekeeping protein. Two representative tumorous tissue samples show downregulation of the protein expression for PTEN; another two tissue sample pairs show upregulation of the protein for PTEN in the tumorous tissue (Figure 6). In one of our samples, the PTEN was nearly in the detection limit while others showed a higher level of protein for PTEN (Figure 6).

IDO protein in the tumorous sediments was upregulated in all four examined sample pairs (Figure 6). Two sample pairs show the same expression changes for both IDO and PTEN. In the other two sample pairs, the IDO expression was upregulated while PTEN expression was downregulation. Although, one of the tumorous tissue’s sediment PTEN protein expression level was nearly at the detection limit.

### 3.4. Detection of IDO in Control (Untreated) and IFN-α2-Treated CAKI-2 and A-498 Human Kidney Cancer Cell Lines

**Figure 7:**
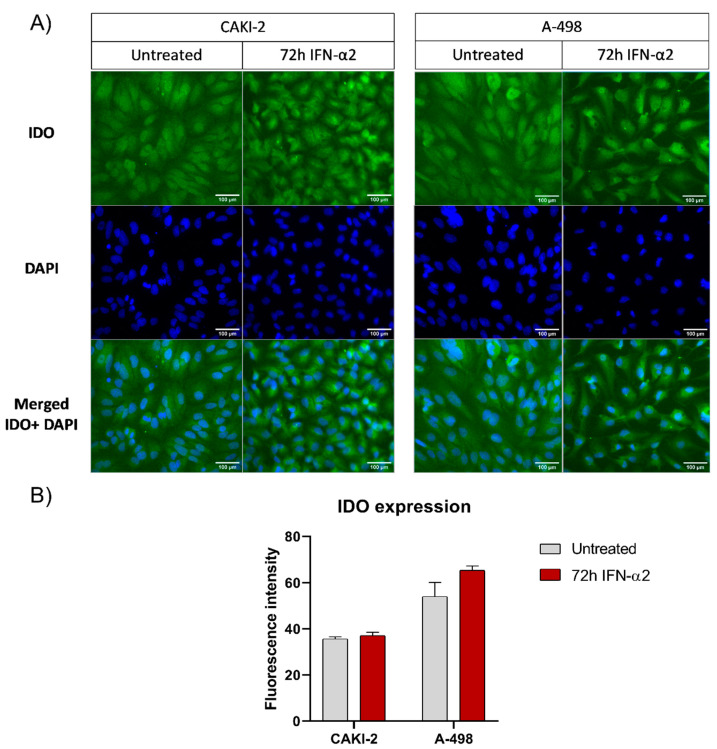
Immunofluorescent labeling of IDO protein in CAKI-2 and A-498 human kidney cancer cell lines (**A**) and the detected fluorescent intensity (**B**). A total of 5 × 10^4^ cells were cultured on microscopic slides and placed in a 6-well plate. Cells were treated with 1000 U/mL IFN-α2 for 72 h. After the treatment, the cells were fixed by methanol and labeled with primary monoclonal antibody against IDO overnight at 4 °C. The next day, the primary antibody was discarded, and the samples were incubated with Alexa 488 conjugated secondary antibody for 1 h at room temperature. DAPI was used for nuclear counterstaining (blue fluorescent signal). The fluorescent signal was detected using a ZEISS Axioscope fluorescent microscope with 40× magnification, the intensity of the IDO expression was evaluated by Fiji ImageJ software 1.53t and normalized to the background.

Treatment with IFN-α2 for 24–72 h induced IDO expression in both CAKI-2 and A-498 human kidney cancer cell lines. IDO expression significantly increased after 24 h of treatment in both cell lines, showing a higher level of induction in A-498 cells compared to CAKI-2 cells. According to the real-time qPCR analysis (Supplementary File for Figure S1), IDO levels gradually decreased by 72 h, approaching those of the untreated control cells, although levels remained slightly elevated even at this later time point. The fluorescent microscopic observation of the interferon-alpha-2-treated CAKI-2 and A-498 cells showed a slightly positive IDO staining. Moreover, both kidney cancer cell lines treated with IFN-α2 exhibited higher expression of IDO compared to the untreated cells. IDO expression after the 72 h treatment with IFN-α2 was elevated in both of the investigated cell lines. These results confirm that the microenvironment of the kidney cancer cells might significantly affect the expression of IDO (Figure 7B).

## 4. Discussion

Patients with kidney tumors diagnosed in time respond well to therapy. The 40% mortality rate is primarily due to the fact that the diagnosis of the disease is made late because of the lack of specific symptoms [4]. In such cases, metastasis may have already developed, at which point surgery or even sunitinib with combined immunotherapy (pembrolizumab, nivolumab, ipilimumab, and nivolumab) cannot help. That is why it is important to recognize the disease in its early phase and to learn about potential biomarkers that can help in precise and early diagnosis [13].

Elevated levels of IDO could result in an immunosuppressive microenvironment around various tumors. Elevated levels could be a prognostic biomarker in multiple cancer types such as colorectal cancer [30,45] or hepatocellular carcinoma [31]. Indoleamine 2,3 dioxygenase is a potential biomarker for various other tumor types such as non-small cell lung cancer, and ovarian cancer [46]. In contrast with other tumor types such as cervical cancer or glioblastoma multiforme, elevated IDO expression is considered a good prognostic factor for RCC patients since they show higher sensitivity to immunotherapies [47]. PTEN is a widely investigated tumor oncogene as it has a key role in cell development through the PI3K/PTEN/AKT pathway [37]. The decreased level or loss of PTEN is a bad prognostic factor in multiple cancer types including RCC [41]. Lower mRNA and protein levels of PTEN can function as a negative prognostic factor in RCC cases as patients with normal or elevated levels of PTEN have a significantly higher survival rate compared to patients with decreased PTEN expression [48].

In our study, mRNA expression for IDO was found to be increased in more than half of the 20 investigated RCC cases. Significantly increased IDO mRNA levels were observed in the tumorous samples (*p* = 0.0261) compared to the healthy tissue samples. Riesenberg et al. published elevated expression of IDO mRNA in RCC in more than 75% of the nearly one hundred investigated cases [32]. The reason for these somehow contradictory results might be the lower number of samples, which is a limitation of our study. Three tumorous samples showed a more than two-times higher IDO mRNA level compared to their healthy pairs. A sample with high IDO expression was identified in an 83-year-old female patient diagnosed with clear-cell renal cell carcinoma (ccRCC), classified as pathological Grade 2 and stage pT1a. In this case, IDO expression was significantly elevated in the tumorous tissue, showing approximately a 13.4-fold increase compared to the adjacent healthy tissue. We assume that specific factors within the tumor microenvironment can markedly influence IDO expression. These may include immune cell infiltration (e.g., T-lymphocytes and dendritic cells), local cytokine production, endothelial cell presence, and hypoxic conditions—factors known to enhance IDO levels and contribute to immune modulation within the tumor [23]. Also, a high expression of IDO (a ~2.45 fold) was detected in another case, a 76-year-old female diagnosed with Grade 2 ccRCC. A sample that was isolated from a 65-year-old patient with Grade 3 ccRCC demonstrated a ~4.65-fold IDO overexpression in a tumorous tissue part compared to an adjacent healthy section. Interestingly, a healthy part of the tissue sample obtained from a 68-year-old male patient with the papillary type of RCC, Grade 2 and pT1a, showed a ~4.6-fold higher IDO level compared to the adjacent tumorous tissue. These few highlighted examples show that the pathological grade and the histological type of RCC might influence the expression of IDO. However, because of the small sample number and also the unequal distribution of the samples by grade, a clear conclusion about the correlation of grades and the IDO expression cannot be drawn. The Western blot analysis also proved the expression of IDO in the sample pairs analyzed.

More than half of the investigated RCC tissue samples showed increased PTEN mRNA expression compared to their healthy renal tissue sample pairs. In two cases, PTEN mRNA expression was around 3-times higher, and one sample showed a much higher expression level. Based on our results, no significant difference could be found between the grades and the expression of IDO/PTEN mRNA. Therefore, no conclusions can be drawn regarding the prognostic value of IDO/PTEN.

From the twenty (80%) investigated tissue sample pairs, IDO and PTEN expression changed in the same way in tumorous sediments compared to the healthy ones. Our results show that 16 out of the 20 examined sample pairs showed strong positive correlation between the mRNA expression of IDO and PTEN. This might indicate that PTEN expression has an influence on the expression of IDO in our samples.

Our experiments with RCC cell lines (A-498; CAKI-2) pointed out that IDO is expressed in the two RCC cell lines, and this is inducible with IFN-α2 treatment. Trott et al. published similar results for IFN-α2 treatment on A-498 and CAKI-1 cell lines [44]. Interferons have commonly been used in renal tumor therapy [49]. IFN-α2 showed significant benefits for patients with metastatic RCC who underwent nephrectomy [50]. A higher IDO expression level is favorable for patients with RCC treated with immunotherapy, but it is not yet known if the interferon treatment is increasing the IDO level or if the IDO level has a significant effect on the response of the patients [49]. IDO has been believed to act as a double-edged sword. In most cancer cases, an elevated IDO level is considered as a bad prognostic factor as it creates an immunosuppressive tumor microenvironment. The healthy cells produce IDO to suppress T-cell-mediated immune responses against self-antigens, fetal antigens, or allogeneic antigens, in different situations. Cancer cells use this mechanism to evade an immune response by overexpressing IDO [51]. However, in RCC cases, an elevated IDO level seems to be favorable for patients who receive immunotherapy. Interactions between the tumor microenvironment and the host immune system influence the response to immunotherapy. CD8+ T cell inflamed tumors can activate various immunosuppressive pathways, such as IDO-1 and/or PD-L1. This suggests the hypothesis that blocking more than one immunosuppressive molecule by combining PD-1/PD-L1 inhibitors with IDO-1 inhibitors may improve the therapeutic response to immunotherapy [33]. Also, untreated CAKI-2 and A-498 cell lines showed mRNA and protein expression of PTEN. No significant IDO induction was observed in the CAKI-2 and A-498 cell lines upon treatment with IFN-α2. However, the results obtained from the quantified IF images show that IFN-α2 induces a certain level of IDO production in the CAKI-2 and A-498 cell lines we examined. According to the literature, it is known that IFN-α2 has only a weak direct effect on IDO induction, and its indirect effect is likely mediated by a smaller by-product of IFN-α2 that stimulates monocytes and lymphocytes in the tumor microenvironment [52]. These, in turn, produce IFN-γ, which triggers significant IDO induction.

Both PTEN and IDO showed overexpression in half of the analyzed tissue sample pairs. In four cases, both IDO and PTEN mRNA levels were decreased in the tumorous tissue. Although the higher IDO level could mean an immunosuppressive tumor microenvironment, studies showed that it is a good prognostic factor as these patients respond better to immunotherapy [33,53]. Significantly higher IDO expression was previously described in PTEN-deficient prostate cancer, but a correlation has never been reported in human RCC [54]. Our results suggest that there might be a positive correlation between the IDO and PTEN expression in kidney tumors, as in 80% of the investigated cases the IDO and PTEN expression showed similar changes in tumorous samples compared to their healthy pairs (Figure S2). The loss of PTEN is usually a bad prognostic factor and frequently occurs in RCC [6]. However, in the samples we studied, it seems that PTEN still shows high expression in the majority of the tumorous samples. According to quantitative PCR analyses from the investigated 20 tumorous and healthy tissue samples, none of them showed a loss in PTEN. The higher PTEN expression in our samples may also be related to the fact that almost all of the examined tissues originate from primary RCC, when PTEN loss has presumably not yet occurred as a result of renal tumorigenesis. Although PTEN is considered to be a tumor suppressor gene, its expression is elevated in multiple cancer types including RCC [55,56]. Thus, we may assume that PTEN has not yet suffered a loss; so, in addition to preserving its tumor suppressor function, we may assume that in the studied samples lower or higher PTEN expression, as a key regulator of the PI3K/AKT pathway, may influence the prognosis of RCC, disease outcomes, and therapeutic strategies [57,58]. From a unique, personalized therapeutic point of view, the results evaluated based on the mRNA expression of IDO/PTEN may also reflect the patient’s possible response to immunotherapy. A previous publication showed that patients with higher IDO expression would react more positively to the therapy combined with IDO inhibitors [53]. The relatively small number of samples used in our study, as well as their non-unified distribution according to tumor grades, allow us to draw only limited conclusions about the correlation of IDO and PTEN.

In RCC, IDO was considered as a potential biomarker; moreover, it shows a negative correlation with the (marker of proliferation Kiel 67) Ki-67 expression in ccRCC [53]. Another study reported a significant negative correlation between PTEN and Ki-67 expression based on data from more than 40 non-small cell lung cancer tissue sample pairs [59]. Our results support the previous results as we found a positive correlation between IDO and PTEN. We suppose the higher mRNA and protein levels of IDO in certain tumor microenvironments might function as a good prognostic factor in RCC, and the elevated PTEN expression might also be a good marker in most tumor cases. Regarding the possible mechanism of the interaction between IDO and PTEN, we might speculate that higher PTEN levels may originate from adjacent cells of the tumor microenvironment e.g., endothelial cells, T cells, etc. Based on a few recent studies, we might assume that IDO released from adjacent cells could trigger a PTEN expression loop (in TREG cells) through the decreased tryptophan level which could lead to cell death and apoptosis [23].

In summary, our results presented here show a positive correlation between PTEN and IDO expression and may help us to better understand the development and pathogenesis of renal cell carcinomas.

## 5. Conclusions

This is one of the first studies where correlation between the expression of mRNA for IDO and PTEN is investigated in ccRCC cases. Significant IDO and PTEN mRNA upregulation was detected when comparing the tumorous tissue samples and the healthy sediments. However, due to the limited number of samples, a meaningful conclusion cannot be drawn regarding the expression of IDO/PTEN in tumorous and adjacent healthy tissues based on the current study. Co-expression of IDO and PTEN in the studied samples suggest that there might be a positive correlation between the IDO and PTEN expression in kidney tumors. It is also assumed that the microenvironment of the kidney cancer cells might significantly affect the expression of IDO. These results help us to understand the possible role of IDO and PTEN in the development of renal carcinoma. However, we are also aware of the limitation of our study because the relatively small sample number allows us to only partly understand the possible role of IDO and PTEN in the pathogenesis of renal cancer. Therefore, in the near future, we would like to extend our investigation, and we are trying to collect a reasonable number of samples. Hopefully, based on the findings in this study investigating 20 healthy and tumorous sample pairs, additional human ccRCC specimens will be able to clarify further questions.

## Figures and Tables

**Table 1:** Clinicopathological data of the 20 patients involved in our study.

Number	Gender	Age	Histology	Grade	TNM
1	Female	52	cc. RCC	3	pT1b
2	Female	79	cc. RCC	2	pT3a pN0
3	Female	59	cc. RCC	3	pT1a
4	Male	67	angioleiomyolipoma	0	0
5	Female	63	cc. RCC	2	pT1a
6	Male	60	Papillary RCC	2	pT1a
7	Female	83	cc. RCC	2	pT1a
8	Female	67	cc. RCC	1	pT1b
9	Male	78	cc. RCC	2	pT1b
10	Female	49	oncocytoma	0	0
11	Female	95	oncocytoma	0	0
12	Male	67	cc. RCC	1	pT1b
13	Female	58	cc. RCC	1	pT1b
14	Female	51	cc. RCC	3	pT3a pN1
15	Male	65	cc. RCC	3	pT3a pN1
16	Male	68	Papillary RCC	2	pT1a
17	Female	76	cc. RCC	2	pT1a
18	Female	57	cc. RCC	2	pT1a
19	Female	65	cc. RCC	3	pT1b
20	Male	83	cc. RCC	2	pT1b

Patients were staged with TNM classification; pT1a: tumor size is less than 4 cm and organ localized; pT1b: tumor size is more than 4 cm, but less than 7 cm, and the tumor is organ localized; pT3a: tumor extends into the renal vein or its segmental branches; ccRCC: clear-cell renal cell carcinoma; pRCC: papillary type of renal carcinoma. N0: lymph node status of the patient is negative and there are no metastases; pN1: micrometastases or metastases in 1–3 axillary lymph nodes.

**Table 2:** Main clinicopathological data with the mRNA expression of IDO and PTEN.

Number	Gender	Age	Relative IDO Expression (ΔΔCp)		Relative PTEN Expression (ΔΔCp)	
1	Female	52	Up	1.45	Up *	3.25
2	Female	79	Down	0.48	Down	0.81
3	Female	59	Up	1.18	Up	1.04
4	Male	67	Up	1.26	Up	1.32
5	Female	63	Up	1.51	Up	1.22
6	Male	60	Up	1.87	Up	1.61
7	Female	83	Up *	13.38	Up *	31.79
8	Female	67	Down	0.62	Down	0.59
9	Male	78	Up	1.56	Up	1.29
10	Female	49	Down	0.78	Up	1.41
11	Female	95	Up	1.21	Down	0.88
12	Male	67	Down	0.23	Down	0.68
13	Female	58	Down	0.42	Down	0.75
14	Female	51	Down	0.30	Up	1.25
15	Male	65	Up *	4.65	Up *	2.94
16	Male	68	Down	0.22	Down	0.68
17	Female	76	Up *	2.44	Up	1.12
18	Female	57	Down	0.92	Up	1.18
19	Female	65	Down	0.79	Down	0.97
20	Male	83	Up	1.30	Up	1.39
Mean ΔΔCp				1.83		2.81

* Overexpression is at least two-fold compared to healthy tissue. Up = upregulation in the tumor tissue compared to the healthy tissue sample; Down = downregulation in the tumor tissue compared to the healthy tissue sample. Mean expression for GAPDH is 21.00 in tumorous and 21.25 in healthy samples, respectively.

## Data Availability

The data presented in this study are available on request from the corresponding author.

## Supplementary Materials

The following supporting information can be downloaded at: https://www.mdpi.com/article/10.3390/cimb47050359/s1.

## Abbreviations

The following abbreviations are used in this manuscript:

IDO	Indoleamine 2,3-dioxygenase
PTEN	Phosphatase and tensin homolog
IFN-α2	Interferon alpha-2
RCC	Renal cell carcinoma
ccRCC	Clear-cell renal cell carcinoma
CA9	Carbonic anhydrase 9
MUC1	The mucin-1
miRNA	MicroRNAs (miRNA)
IDO2	Indoleamine 2,3-dioxygenase-2
TDO	Tryptophan-2,3-dioxygenase
IFN-γ	Interferon gamma
ATCC	American Type Culture Collection
IMDM	Iscove’s Modified Dulbecco’s Medium
BCA	Bicinchoninic Acid
SDA-PAGE	Dodecyl sulfate-polyacrylamide gel electrophoresis
PVDF	Polyvinylidene fluoride
HRP	Horseradish peroxidase
ROI	Region of Interest
