# Supplementary material for: Evaluation of the Expression of IDO and PTEN in Human Kidney Cancer
Source: Curr Issues Mol Biol. 2025 May 13;47(5):359. doi: 10.3390/cimb47050359 (PMC12110602; doi:10.3390/cimb47050359)
Supplement: Supplementary file 1 [file cimb-47-00359-s001.zip › cimb-3598070-supplementary.pdf]

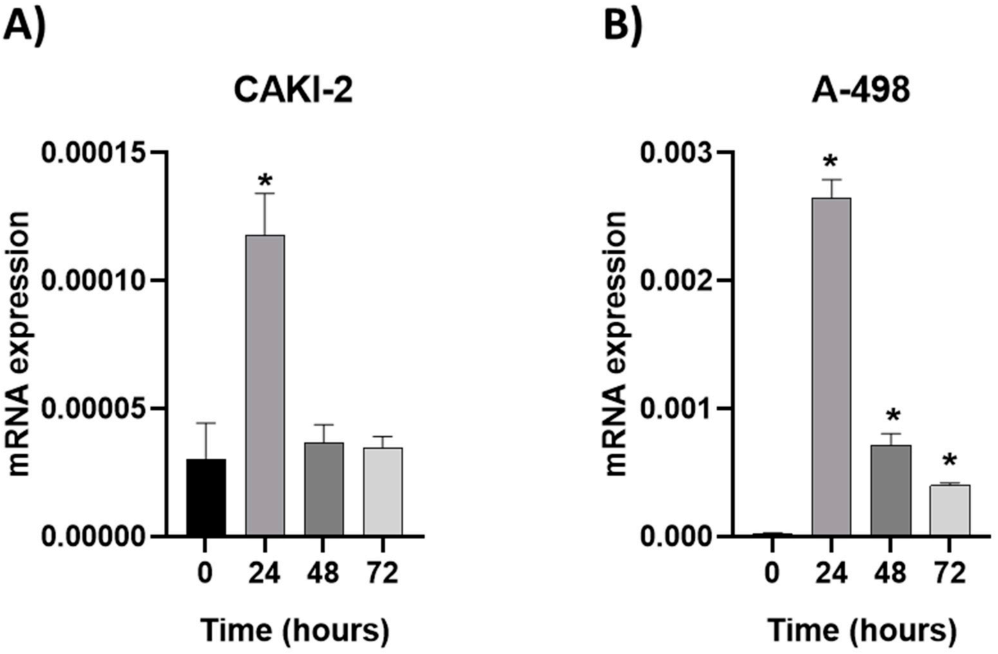

Supplementary to Figure S1. qPCR analysis of mRNA for IDO expression in CAKI-2 (A) and A-498 (B) renal cancer cell lines after IFN-α2 treatment. Cells were treated with 1000 U/ml IFN-α2 for 72 h. The experiment was carried out as described in the *Materials and Methods* section (subsection 2.2-2.4). 40 µg of cDNA was used in each PCR reaction and GAPDH served as a housekeeping gene. Statistically significant differences have been found using the two-way ANOVA test with Sidak multiple comparison test ($p < 0.05$).

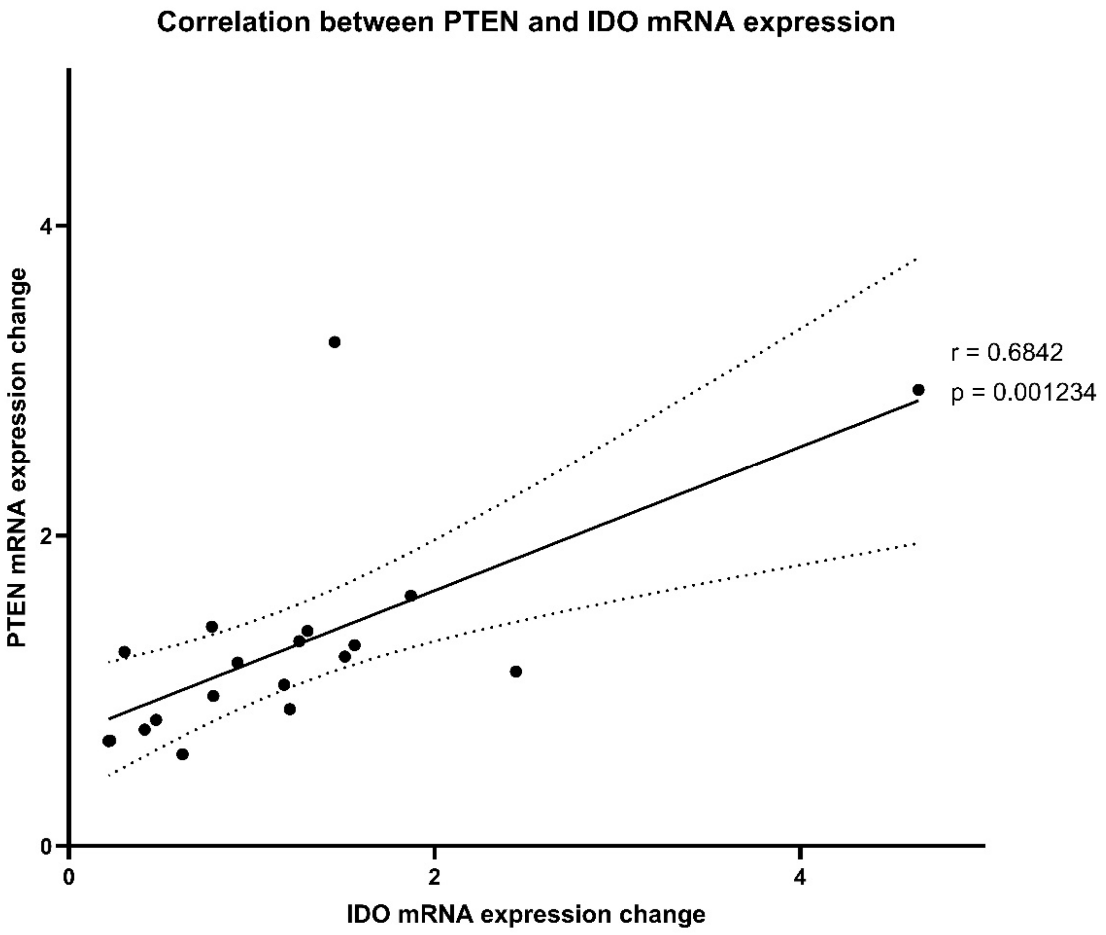

Supplementary Figure S2: IDO and PTEN mRNA expression changes in 19 tumorous and healthy kidney tissue pairs; patient number 7 was considered as an outlier. Correlation analysis was made using the Spearman-method. Positive correlation can be observed (r = 0.6842) between the IDO and PTEN mRNA expression.
